# Expression analysis of three isoforms of hyaluronan synthase and hyaluronidase in the synovium of knees in osteoarthritis and rheumatoid arthritis by quantitative real-time reverse transcriptase polymerase chain reaction

**DOI:** 10.1186/ar1223

**Published:** 2004-09-22

**Authors:** Mamoru Yoshida, Shigaku Sai, Keishi Marumo, Takaaki Tanaka, Naoki Itano, Koji Kimata, Katsuyuki Fujii

**Affiliations:** 1 Department of Orthopaedic Surgery, The Jikei University School of Medicine, Tokyo, Japan; 2 Institute for Molecular Science of Medicine, Aichi Medical University, Aichi, Japan

**Keywords:** arthritis, hyaluronan, hyaluronan synthase, hyaluronidase, synovium

## Abstract

Hyaluronan is a major molecule in joint fluid and plays a crucial role in joint motion and the maintenance of joint homeostasis. The concentration and average molecular weight of hyaluronan in the joint fluids are reduced in osteoarthritis and rheumatoid arthritis. To elucidate the underlying mechanism, we analyzed the message expression of three isoforms of hyaluronan synthase and hyaluronidase from knee synovium, using real-time reverse transcriptase polymerase chain reaction. Synovia were obtained from 17 patients with osteoarthritis, 14 patients with rheumatoid arthritis, and 20 healthy control donors. The message expression of hyaluronan synthase-1 and -2 in the synovium of both types of arthritis was significantly less than in the control synovium, whereas that of hyaluronidase-2 in the synovium of both arthritides was significantly greater than in the control synovium. The decreased expression of the messages for hyaluronan synthase-1 and -2 and/or the increased expression of the message for hyaluronidase-2 may be reflected in the reduced concentration and decreased average molecular weight of hyaluronan in the joint fluids of patients with osteoarthritis and rheumatoid arthritis.

## Introduction

High-molecular-weight (HMW) hyaluronan (average molecular weight 6–7 × 10^6 ^Da) is a major component of synovial joint fluids [1-5]. It physically acts as a viscous lubricant for slow joint movements, such as walking, and as an elastic shock absorber during rapid movements, such as running [6]. HMW hyaluronan has a variety of biologic effects on cells *in vitro*, such as: the inhibition of prostaglandin E_2 _synthesis and the release of arachidonic acid induced by interleukin-1 from cultured fibroblasts [7,8]; protection against proteoglycan depletion and cytotoxicity induced by oxygen-derived free radicals, interleukin-1, and mononuclear-cell-conditioned medium [9,10]; and the suppression of phagocytosis, of locomotion, and of enzyme release by leukocytes and macrophages [11-14]. HMW hyaluronan has been shown to suppress the degradation of cartilage matrix induced by fibronectin fragments [15,16] and cytokines [17]. Moreover, it has been shown to relieve joint pain by masking free nerve ending organelles in animal experiments [18,19]. Hence, it is suggested that HMW hyaluronan is an indispensable component in the maintenance of articular joint homeostasis. Reductions in the concentration and average molecular weight of hyaluronan in knee synovial fluids from patients with osteoarthritis (OA) or rheumatoid arthritis (RA) have been reported [2,3,20-25]. These reductions indicate hyaluronan's involvement in the pathogenesis of these joint disorders and are reflected in the pathological changes of hyaluronan metabolism.

Hyaluronan is synthesized by hyaluronan synthases (HASs) located at the plasma membrane of cells [26]. Three HAS isoforms, encoded by three distinct genes, are expressed in human knee synovium [27]. It is believed that joint fluid hyaluronan is mainly supplied from type B cells – proper synoviocytes – of the synovial lining [2-5,28]. Little is known about hyaluronan catabolism in synovial fluid. It is thought that hyaluronan is eliminated by the lymphatic or vascular system after fragmentation by an unknown process [29] or that macrophagic type A cells of the synovial lining absorb and digest hyaluronan, because type A cells have many vesicles and vacuoles containing lysosomal enzyme – such as nonspecific esterase, acid phosphatase, and cathepsins B, D, and L – and type A cells are active in the uptake of substances in synovial fluids [28]. Hyaluronidase, which specifically degrades hyaluronan, is a lysosomal enzyme. Among five homologous isozymes in humans, hyaluronidase-1, -2, and -3 are thought to be expressed in synovium and involved in hyaluronan degradation, since hyaluronidase-4 is a chondroitinase and hyaluronidase-5, the sperm-specific enzyme PH-20, is specifically expressed in sperm [30]. The messages of hyaluronidase-1, -2, and -3 are expressed in chondrocyte monolayer cultures and in extracts of fresh human cartilage [31].

In the present study, we investigated message expression levels for three isoforms of HAS and hyaluronidase in knee synovium obtained from control donors and patients with OA or RA, by quantitative reverse transcriptase polymerase chain reaction (RT-PCR), in order to confirm whether message levels differed.

## Materials and methods

### Materials

An RNeasy kit was purchased from QIAGEN KK (Tokyo, Japan). Primer Express computer software, gene-specific primer pairs and probes, TaqMan Gold RT-PCR reagents without controls, Pre-Developed TaqMan assay reagents of endogenous control human beta-actin, and a 7700 sequence detector were purchased from Perkin-Elmer Corp (Norwalk, CT, USA). The Hyaluronate-Chugai test kit was from Chugai Pharmaceutical Corp (Tokyo, Japan).

### Patients and controls

Baseline data for patients with OA or RA and for control donors from whom synovial samples were obtained are summarized in Table 1. Two of us (MY and SS), both physicians, clinically diagnosed OA and diagnosed RA according to the criteria of the American Rheumatism Association. Pharmacological treatment before sampling was limited to analgesics or nonsteroidal anti-inflammatory drugs in all study subjects. Rheumatoid arthritis patients were classified in stage II or stage III, and class II grade according to the Steinbrocker classification. The radiographic grades of all knee joints were determined on frontal views of the tibiofemoral joints according to the radiographic atlas recommended by the Osteoarthritis Research Society [32]. Grade B radiographic appearance, corresponding to grade 1 of the Larsen grading system, is defined by the presence of grade 1 joint space narrowing combined with osteophytes, or of grade 2 or 3 joint space narrowing. Control synovial samples were obtained from donors who had no intra-articular pathologic findings under arthroscopy at second-look observations after partial meniscectomy or from donors who complained of knee pain of unknown etiology but who had no intra-articular pathologic findings under arthroscopy on routine examination. The control synovium donors were significantly younger than the patients with OA or RA (*P *< 0.01).

### Sampling of synovial tissues and isolation of total ribonucleic acid

We obtained informed consent from all the study subjects and approval by the university ethical committee and the institutional review board. Synovial tissue samples were obtained from the central area of the suprapatellar pouches of the knees during arthroscopic examination, arthroscopic surgery, or open surgery performed in a hospital of the Jikei University School of Medicine. After subsynovial or fatty tissues were macroscopically resected from the obtained samples, all synovial samples were immediately frozen with liquid nitrogen and stored at -80°C. The total RNA of each sample was isolated using an RNeasy kit.

### Analysis of hyaluronan in joint fluid

Joint fluid was aspirated from the knee immediately before an examination or surgery and was stored at -80°C. Joint fluid was obtained from 10 healthy control donors, 10 patients with OA, and 10 with RA. Hyaluronan concentration in joint fluid was measured by a sandwich binding protein assay using a Hyaluronate-Chugai test kit [33]. The molecular weight of hyaluronan was calculated from the intrinsic viscosity of hyaluronan in fluid, which was measured with a capillary viscometer [34] after pronase treatment. This method was chosen because it is more precise than HPLC analysis for the measurement of the average molecular weight of HMW hyaluronan.

### Analysis of message expression by quantitative real-time RT-PCR

Message expression in the synovium of knees and the relative differences in message levels between the control group and patients with OA or RA were determined by real-time RT-PCR in accordance with the manufacturer's instructions and reported methods [35-39]. The gene-specific PCR oligonucleotide primer pairs and gene-specific oligonucleotide probes labelled with a reporter fluorescent dye (FAM) at the 5'-end and a quencher fluorescent dye (TAMURA) at the 3'-end were designed using the Primer Express computer software for HAS-1, -2, and -3 and hyaluronidase-1, -2, and -3 genes. For the HAS-2 probe, a minor groove binder probe was used to achieve an optimal melting temperature, because a suitable site for the regular probe was not found in the DNA sequences of HAS-2 (Table 2). A minor groove binder is an enhancer of the probe's melting temperature. Total RNA (200 ng for each) was added to a 50 μL RT-PCR reaction buffer containing 0.2 mmol/L deoxynucleotide triphosphates, 1.5 mmol/L MgSO_4_, 2.5 μmol/L random hexamers, 0.1 U/mL MultiScribe reverse transcriptase, 0.1 U/μL AmpliTaq Gold DNA polymerase, 900 nmol/L concentration of PCR primer pairs, 200 nmol/L concentration of the corresponding probe, and 2.5 μL Pre-Developed TaqMan assay reagents of endogenous control human β-actin, which contained β-actin-specific primers and probes labeled with a different reporter fluorescent dye (VIC). RT-PCR was carried out in a 96-well plate under the following conditions: one cycle at 50°C for 2 minutes to activate the uracil *N*-glycosylase, one cycle at 60°C for 30 minutes, one cycle at 95°C for 5 minutes, and 50 cycles at 95°C for 20 seconds and 60°C for 1 minute. The fluorescence energy emitted from the reporter dye without a quencher was monitored directly by a 7700 sequence detector in real time when the annealed probes were broken by DNA polymerases during the polymerization period. The threshold cycle numbers (*C*_T_), from which the logarithmic amplification phase of the PCR reaction started, were determined simultaneously for the messages of both target gene and β-actin gene in the same sample tube when the intensity of the reporter fluorescent signal reached 10 times the standard deviation of the baseline of fluorescent signal intensity. The *C*_T _value of the β-actin message was used as an internal standard.

When the target messages were detected in both the control samples and the OA or RA samples by RT-PCR, the ratio for the amount of the message expressed in control samples to the amount of the message expressed in OA or RA samples was determined as a relative expression level. Relative expression levels of the target messages were calculated as follows: the Δ*C*_T _of each target message was obtained by subtracting the *C*_T _of β-actin message from the *C*_T _of each target message in the same RNA sample. The Δ*C*_T _values of the same target message between the control and OA or RA groups were analyzed statistically. When these Δ*C*_T _values were significantly different (*P *< 0.01), the averageΔ*C*_T _value of each target message was calculated from all the Δ*C*_T _values. The ΔaverageΔ*C*_T _value of each message was obtained by subtracting the averageΔ*C*_T _of control samples from the averageΔ*C*_T _of OA or RA samples. Finally, the relative expression level of each target message was determined using the formula: relative expression level = 2^-ΔaverageΔ*C*T^

### Statistical analysis

Statistical analysis was with Wilcoxon's matched-pairs signed rank test. A probability value of <0.01 was considered statistically significant.

## Results

### Concentration and average molecular weight of hyaluronan in synovial fluid

The concentration and average molecular weight of hyaluronan in the synovial fluid of OA or RA patients were significantly lower than those of control donors (Table 3).

### Expression profile of hyaluronan synthase isoform messages

**Figure 1:**
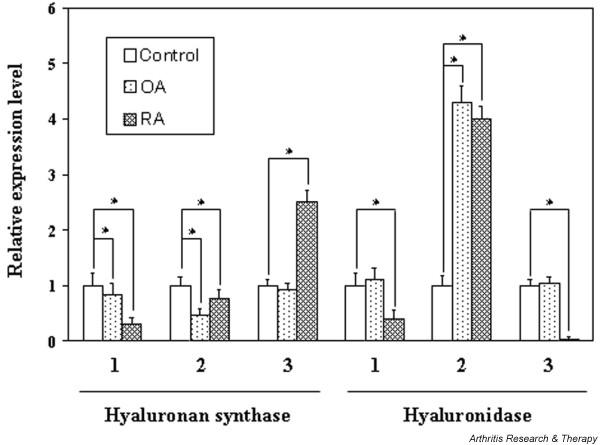
Relative expression levels of the messages for hyaluronan synthase-1, -2, and -3 and hyaluronidase-1, -2, and -3 in the synovium of knees in osteoarthritis (OA) and rheumatoid arthritis (RA). Total RNA was isolated from knee synovium and expression levels of the messages were relatively quantified by real-time reverse transcriptase polymerase chain reaction. The expression levels of the messages in OA and RA are expressed by longitudinal bars relative to those of the control value expressed as 1.0. The lines outside the bars represent the standard deviation of the change in threshold cycle numbers (Δ*C*_T_) corrected with *C*_T _values of β-actin message used as an internal standard. * *P *< 0.01.

Expressed messages for all three HAS isoforms were detected in all synovial samples. The expression of the messages for HAS-1 and HAS-2 was significantly less in the synovium of OA than in the control synovium (83% and 48% of the respective control values), whereas no significant difference was observed for HAS-3 message expression. HAS-1 and HAS-2 message expression in RA synovium was significantly less than in control synovium (30% and 77% of the respective control values), while the expression of HAS-3 message was significantly greater than that in the control synovium (250% of the control value) (Fig. 1).

### Expression profile of hyaluronidase isozyme messages

Message expression of all three hyaluronidase isozymes was detected in all synovial samples. Message expression for hyaluronidase-2 in OA synovium was significantly increased (to 430% of that in control synovium), while no significant differences were observed for hyaluronidase-1 and hyaluronidase-3 message expression. The expression of the message for hyaluronidase-2 was significantly greater (400% of the control value), while the expression of messages for hyaluronidase-1 and hyaluronidase-3 was significantly decreased in RA synovium (to 40% and 3% of the respective control values) (Fig. 1).

## Discussion

The present study showed that HAS-1 and HAS-2 message expression was decreased in OA and RA synovium. This finding suggests that the protein expression of HAS-1 and -2 is decreased, as it has been reported that message levels are correlated with HAS protein levels and with the production of hyaluronan in cultured cells [40]. It has been suggested that the expression level of HAS proteins and their synthetic activities regulate the total volume of hyaluronan produced by cells, because detergent-purified HAS proteins alone can synthesize hyaluronan and no associated proteins or components are necessary for hyaluronan synthesis *in vitro *[41]. HAS activity of stable transfectants of HAS-2 is approximately 1.2 times that of HAS-1 or HAS-3 [42]. Stable transfectants of HAS-1 and HAS-3 produce hyaluronan with a broad size distribution (molecular weights of 2 × 10^5 ^Da to approximately 2 × 10^6 ^Da), whereas stable transfectants of HAS-2 produce hyaluronan with a broad size distribution that ranges higher (average molecular weight of >2 × 10^6 ^Da) [41]. Among HAS isoforms, the predominant message expressed in human knee synovium is HAS-1 [27]. Therefore, synovial production of hyaluronan, including HMW hyaluronan, may be decreased in OA or RA. A reduced production of HMW hyaluronan may be involved in the pathogenesis of these joint disorders, since HMW hyaluronan has important physical and biologic functions, as described in the Introduction.

An age-associated change in synoviocyte population revealed that the number of type B cells was significantly decreased in older animals, although this was not confirmed in humans [28]. The message levels of all three HAS isoforms were not uniformly decreased in the knee synovium of OA or RA patients, even though the patients were significantly older than the control donors. Hence, it is unclear whether the different expression profiles of HAS messages in the controls, OA and RA patients are attributable to age-associated change, to physical senility, or to a pathologic factor specific for arthritic joint disorders.

Hyaluronidase activity was detected in human knee synovial fluid of OA or RA patients when the assay was performed at the acidic pH of 4.5, but not at pH 5.0–7.0 [43]. Hyaluronidase-1 may be present in the fluids, because it is a major isozyme in plasma and urine and is unable to bind hyaluronan at neutral pH [30]. We suggest that soluble forms of hyaluronidases in synovial fluids are not involved in the direct digestion of hyaluronan in joint fluids, because a neutral pH is maintained in synovial fluids, and so hyaluronidase-1 may function only within lysosomes.

Hyaluronidase-2 is linked to the outer cell membrane by a glycosylphosphatidyl-inositol (GPI) anchor and it digests hyaluronan to intermediate-sized fragments of approximately 20 kDa, while hyaluronidase-1 digests hyaluronan to tetrasaccharides [30]. A process of hyaluronan catabolism in somatic cells proposed in the review literature [30] is that hyaluronan is taken up into unique endocytic vesicles by an unknown mechanism and is digested into 20-kDa fragments by hyaluronidase-2 located in vesicles at an acidic pH; subsequently, the fragments are transported into lysosomes, where hyaluronidase-1 and two exoglycosidases digest hyaluronan into monosaccharides. The present study showed that the message expression of hyaluronidase-2 in the synovium of OA and RA was approximately four times that in the control synovium. This finding suggests that in OA and RA, the protein expression of hyaluronidase-2 in the synovium is increased and the hyaluronan digestion by hyaluronidase-2 is accelerated.

Little is known about hyaluronidase-3. Strong hybridization expression patterns are found in mammalian testis and bone marrow [30]. Hyaluronidase-3 message expression was detected in synovium in the present study. This isozyme may work only in the lysosomes, as does hyaluronidase-1 [30]. The expression level in RA synovium was significantly lower than in OA or control synovium. The reduction in message expression may be due to the different cellular populations found in OA versus RA, since many inflammatory cells such as granulocytes or lymphocytes appeared in RA synovium.

Joint fluid hyaluronan concentration is determined by the production volume of hyaluronan, the elimination volume of hyaluronan from the joint, and the total volume of joint fluid. The production of hyaluronan in OA or RA may be decreased because of the reduced expression of HAS-1 and -2 messages. The elimination volume of hyaluronan may be increased by the elevated expression of hyaluronidase-2, because hyaluronidase-2 digests hyaluronan in the endosome after uptake of hyaluronan into cells [30]. Hence, it is thought that the decreased expression of HAS-1 and -2 and/or the increased expression of hyaluronidase-2 are among the causes leading to the reduced hyaluronan concentration in OA or RA synovial fluid.

The average molecular weight of hyaluronan in synovial fluid is determined by the molecular weights of hyaluronan produced and hyaluronan digested in the fluid. The average molecular weight of newly produced hyaluronan may be reduced by the decreased expression of HAS-2, because, of the three HAS isoenzymes, HAS-2 synthesizes the highest-molecular-weight hyaluronan [42]. The decreased expression of HAS-2 may be one of the causes for the reduced average molecular weight of hyaluronan in joint fluid. Moreover, there may be a mechanism whereby HMW hyaluronan is digested into low-molecular-weight hyaluronans in synovial fluid, since the average molecular weight of hyaluronan in OA or RA fluid is lower than that of hyaluronan synthesized by HAS-1 or -3.

HAS-3 message expression was increased in RA synovium, although hyaluronan concentration was reduced. The increased expression of HAS-3 message may be due to the increased number of inflammatory cells invading the pannus tissue (which is inflammatory and proliferative granular synovial tissue specific for RA), since a high expression level of HAS-3 message in inflammatory cells was observed in another study by two of us (NI and KK). It is supposed that the hyaluronan produced by inflammatory cells does not diffuse into the joint cavity and that it surrounds cells, protecting them or aiding their migration, because it has been reported that pannus tissue with inflammatory cells contains a greater amount of hyaluronan than is found in OA or traumatic injury [44].

## Conclusion

Message expression for three isoforms of hyaluronan synthase and hyaluronidase in knee synovium differs in OA or RA from that in healthy controls. Differential expression of hyaluronan synthases and/or hyaluronidases may be reflected in the pathological metabolism of hyaluronan in the knee synovial fluid of patients with OA or RA.

## Competing interests

None declared.

## Abbreviations

HAS-1/-2/-3 = hyaluronan synthase-1/-2/-3; HMW = high-molecular-weight; HPLC = high-performance liquid chromatography; OA = osteoarthritis; PCR = polymerase chain reaction; RA = rheumatoid arthritis; RT-PCR = reverse transcriptase polymerase chain reaction; SD = standard deviation.

## Figures and Tables

**Table 1:** Baseline data for subjects with osteoarthritis (OA) or rheumatoid arthritis (RA) or without arthritis

		**Age (years)**		
			
**Subjects**	**Number (male/female)**	**Mean ± SD**	**Range**	**Radiographic grade^a^**
With OA	17 (9/8)	64.77 ± 9.02	45 – 83	B
With RA	14 (2/12)	60.55 ± 12.05	35 – 74	B
Controls	20 (11/9)	37.25 ± 7.59	29 – 59	Normal

^a^Determined on x-ray frontal views of the tibiofemoral joints in accordance with the radiographic atlas recommended by the Osteoarthritis Research Society [32]. SD, standard deviation.

**Table 2:** Sequences of the gene-specific oligonucleotide primers and probes for real-time reverse transcriptase polymerase chain reaction

**Primer and probe**		**Sequence**	**Position**
HAS-1	855F	GACTCCTGGGTCAGCTTCCTAAG	855 – 877
	995R	AAACTGCTGCAAGAGGTTATTCCT	995 – 972
	probe	TATCCTGCATCAGCGGTCCTCTAGGC	940 – 965
HAS-2	20F	CTATGCTTGACCCAGCCTCATC	20 – 41
	149R	ACACTGCTGAGGAATGAGATCCA	149 – 127
	MGB probe^a^	AGATGTCCAGATTTTA	96 – 111
HAS-3	888F	TGTCCAGATCCTCAACAAGTACGA	888 – 911
	1005R	AATACACTGCACACAGCCAAAGTAG	1005 – 981
	probe	TCATGGATTTCCTTCCTGAGCAGCGT	913 – 938
HYAL-1	1561F	AGTGGTGCTCTGGGTGAGCT	1561 – 1580
	1667R	TGGTCACGTTCAGGATGAAGG	1667 – 1647
	probe	CCAAGGAATCATGTCAGGCCATCAAGG	1596 – 1622
HYAL-2	1069F	CGCAGCTGGTGTCATCCTCT	1069 – 1088
	1159R	CAGCAGCCGTGTCAGGTAATC	1159 – 1139
	probe	TACACCACAAGCACGGAGACCTGCC	1103 – 1127
HYAL-3	1130F	GCCTCACACACCGGAGATCT	1130 – 1149
	1202R	GCTGCACTCACACCAATGGA	1202 – 1183
	probe	TCCTGTCCCAGGATGACCTTGTGCA	1157 – 1181

^a^Minor groove binder (MGB) enhances the melting temperature of the probe (see Materials and methods). HAS, hyaluronan synthase; HYAL, hyaluronidase.

**Table 3:** Concentration and molecular weight (mean ± standard deviation) of hyaluronan in synovial fluid of patients with osteoarthritis (OA) or rheumatoid arthritis (RA) and in controls

**Subjects**	**Concentration (mg/mL)**	**Molecular weight (×10^4 ^Da)**
Controls	3.1 ± 0.4	365 ± 52
OA	1.2 ± 0.2*	212 ± 37*
RA	0.8 ± 0.3*	163 ± 33*

* *P *< 0.01 in comparison with controls.
